# In Vitro Antimalarial and Toxicological Activities of *Quercus infectoria* (Olivier) Gall Extracts

**DOI:** 10.21315/mjms2020.27.4.4

**Published:** 2020-08-19

**Authors:** Nik Nor Imam Nik Mat Zin, Mira Nabila Mohamad, Keusar Roslan, Sazeli Abdul Wafi, Nurul I’zaaz Abdul Moin, Azamuddin Alias, Yusmazura Zakaria, Nurhidanatasha Abu-Bakar

**Affiliations:** 1 School of Health Sciences, Universiti Sains Malaysia, Kubang Kerian, Kelantan, Malaysia; 2 Pengiran Anak Puteri Rashidah Sa’adatul Bolkiah Institute of Health Sciences, Universiti Brunei Darussalam, Brunei Darussalam; 3 Department of Biomedical Sciences, Universiti Islam Antarabangsa Malaysia, Kuantan, Pahang, Malaysia

**Keywords:** Quercus infectoria, antimalarial activity, toxicological activity, malarial SYBR Green I fluorescencebased assay, brine shrimp lethality test, haemolytic assay, 3-[4,5-dimethylthiazol-2-yl]-2,5 diphenyl tetrazolium bromide assay

## Abstract

**Background:**

The spread of *Plasmodium falciparum* resistance in common antimalarial drugs, including artemisinin-based combination therapies, has necessitated the discovery of new drugs with novel mechanisms of action. In the present study, the in vitro antimalarial and toxicological activities of acetone, methanol, ethanol and aqueous extracts of *Quercus infectoria* (*Q. infectoria*) galls were investigated.

**Methods:**

The extracts were assessed for the antimalarial potential using a malarial SYBR Green I fluorescence-based (MSF) assay, while the toxicity was screened by using brine shrimp lethality test (BSLT), haemolytic assay, and cytotoxicity assay against normal embryo fibroblast cell line (NIH/3T3) and normal kidney epithelial cell line (Vero).

**Results:**

The acetone extract showed the highest antimalarial activity (50% inhibitory concentration, IC_50_ = 5.85 ± 1.64 μg/mL), followed by the methanol extract (IC_50_ = 10.31 ± 1.90 μg/mL). Meanwhile, the ethanol and aqueous extracts displayed low antimalarial activity with IC_50_ values of 20.00 ± 1.57 and 30.95 μg/mL ± 1.27 μg/mL, respectively. The significant antimalarial activity was demonstrated in all extracts and artemisinin (*P* < 0.05). All extracts were non-toxic to brine shrimps (50% lethality concentration, LC_50_ > 1000 ppm). Furthermore, no occurrence of haemolysis (< 5%) was observed in normal erythrocytes when treated with all extracts compared to Triton X-100 that caused 100% haemolysis (*P* < 0.05). The acetone and methanol extracts were non-toxic to the normal cell lines and statistically significant to artemisinin (*P* < 0.05).

**Conclusion:**

Taken together with satisfactory selectivity index (SI) values, the acetone and methanol extracts of *Q. infectoria* galls could serve as an alternative, promising and safe antimalarial agents.

## Introduction

Malaria remains as one of the devastating infectious parasitic diseases, particularly in tropical and subtropical areas of Asia, Africa and Central and South America (1). World Health Organization (WHO) reported an increasing trend of malaria in 2017, with an estimated 219 million cases worldwide, and 2 million more cases than in 2016 (1). The disease continues to threaten pregnant women and children under 5 years of age, especially in sub-Saharan African and has resulted in nearly half-million deaths worldwide (1). *Plasmodium falciparum* (*P. falciparum*), the most virulent malaria parasite, accounted for 99.7% of the estimated cases in the African region, and more than 60% in Southeast Asia, Eastern Mediterranean and Western Pacific, which caused the majority of deaths and recorded the most severe clinical manifestations (1).

Malaria control is currently dependent on the elimination of *Anopheles* mosquito breeding sites using insecticides, prevention of mosquito-human contact with insecticide-impregnated bed nets and effective case management (1, 2). Malaria case management depended heavily on antimalarial drugs, mainly artemisinin-based combination therapies (ACTs) to treat patients with uncomplicated or severe *P. falciparum* cases (1, 3). ACTs, which are co-formulations of fast-acting, highly-potent artemisinin and a slow-acting, less-potent partner drug, are prescribed first-line treatments for malaria (3, 4). Unfortunately, *P. falciparum* resistance to artemisinin has emerged in several locations in the Greater Mekong subregions in Southeast Asia (5, 6). This could significantly jeopardise the progress accomplished in this region and contribute to an increase in the burden of disease in other endemic countries. Given this bottleneck, there is an urgent need to discover new antimalarial agents, especially from natural resources.

*Quercus infectoria* (*Q. infectoria*) Olivier galls, mainly found in Asia, is developed as a consequence of infections of the plant’s shoots by *Cynips gallae tinctoriae* wasps. It is reported to possess antibacterial (7–9), candidacidal (7), antiviral (10), antioxidant (11) and anti-inflammatory properties (12). The galls have also demonstrated in vitro and in vivo effects against several parasites (11, 13–15). This plant has been used frequently by old folks to reduce high fever (16, 17), which is one of the clinical symptoms of malaria (18); hence, *Q. infectoria* galls might possess antimalarial activity. It is also imperative to investigate its possible toxicity, which may provide greater assurance of the safety of this plant in humans.

The in vitro antimalarial activity of four different extracts of *Q. infectoria* galls against a chloroquine-sensitive 3D7 strain of *P. falciparum* was evaluated in this study. All extracts were also tested against brine shrimps, normal erythrocytes and normal cell lines. The selectivity index (SI), in which the antimalarial activity was compared with the cytotoxicity, was also assessed.

## Methods

### Plant Material

*Q. infectoria* galls (Family: Fagaceae) were purchased from a herbal store in Kota Bharu, Kelantan, Malaysia. The galls were identified based on their physical appearances (19) and authenticated at the Natural Medicinal and Product Centre, Universiti Islam Antarabangsa Malaysia (PIIUM 0229-1).

### Extraction of the Plant Material

Acetone, methanol, ethanol and aqueous extractions were performed using a modified method by Baharuddin et al. (7). The galls were washed, dried and pulverised before dissolved, and macerated in respective solvents at a ratio of 100 g of dried crude powder per 500 mL of absolute solvent for 72 h in a water bath at 50 °C. The extracts were filtered using Whatman filter papers (No. 1). The acetone, methanol and ethanol filtrates were concentrated using a rotary evaporator at 55 °C. The resulting pellets were pounded to dryness at 50 °C for two days to produce powdery and brown crude extracts. The aqueous filtrate was concentrated using a rotary evaporator at 80 °C and the resulting pellet was freeze-dried at −50 °C under vacuum to produce a fine crystal-like crude aqueous extract. The crude extracts were weighed and stored in sealed vials at 4 °C for further use.

### In Vitro Antimalarial Assay

#### In vitro culture of P. falciparum

Chloroquine-sensitive *P. falciparum* (3D7 strain) was provided by the Institute for Research in Molecular Medicine (INFORMM), Universiti Sains Malaysia (USM) and maintained up to 10 mL, mixed and centrifuged. The cell pellet was resuspended in a 1× PBS solution in culture flasks containing complete culture medium (CCM) and washed type O^+^ human erythrocytes at 2% haematocrit using a modified protocol from Mohd-Zamri et al. (20). CCM consists of RPMI 1640 medium with GlutaMAX and 25 mM HEPES (Gibco, Waltham, Massachusetts, USA) enriched with 0.2% glucose (w/v), 50 μg/mL hypoxanthine (Sigma Aldrich, Missouri, USA), 25 μg/mL gentamicin (Duopharma) and 0.25% Albumax II (w/v; Gibco, Waltham, Massachusetts, USA). Cultures were incubated at 37 °C in a humidified atmosphere of 5% CO_2_ and routinely maintained every 2–3 days. When the parasites were mainly at the ring stage (5% parasitaemia) upon confirmation by Giemsa-stained thin blood smears, they were synchronised by sorbitol treatment at a ratio of 100 μL of cell pellet per 1000 μL of 5% D-sorbitol (w/v; Sigma Aldrich, Missouri, USA) to kill mature stage parasites (trophozoite and schizont stages) (21). Synchronised ring stage parasite-infected erythrocytes (~2 h post-synchronisation) were used in the subsequent anti-malarial assay.

## Malarial SYBR Green I Fluorescence-Based Assay

The antimalarial activity of the extracts was assessed via a malarial SYBR Green I fluorescence-based (MSF) assay (22). The extracts were dissolved in 100% dimethyl sulfoxide (DMSO) to produce stock solutions of 300 mg/mL. The stock solutions were subsequently diluted with CCM at 10 concentrations of two-fold dilutions into a 96-well microtiter plate. Aliquots of 20 μL of extract concentrations were transferred into another 96-well microtiter plate. A 180 μL suspension of synchronised ring stage-infected erythrocytes (2% parasitaemia, 2% haematocrit) was added to each well. The final concentration of DMSO in the medium was < 1%. Artemisinin (Sigma Aldrich, Missouri, USA) was used as a standard control, infected erythrocytes devoid of the extracts as a negative control and 100% DMSO as a positive control. The plates were incubated for 48 h at 37 °C in 5% CO_2_. After 48 h incubation, aliquots of 180 μL of the cell suspensions were dispensed in a new 96-well microtiter plate containing 20 μL of 20× SYBR Green I solution (Invitrogen, Waltham, Massachusetts, USA), wrapped in aluminium foils and incubated for 1 h at room temperature (23). The total fluorescence (TF) signal was measured with a microplate reader at the excitation and emission wavelengths of 490 nm and 530 nm, respectively. The percentage of parasite inhibition of each concentration was calculated as follows:

$$\text{Parasite inhibition }(\%)=\frac{\text{TF }(\text{test sample}-\text{blank})}{\text{TF }(\text{negative control}-\text{blank})}\times 100$$

The mean of three half-maximal inhibitory concentration (IC_50_) values of the extracts was determined by using probit regression analysis with GraphPad Prism software (Version 6). All tests were conducted in triplicate on three different occasions.

### Brine Shrimp Lethality Test

The preliminary assessment of the toxicological potential of the extracts was performed using a brine shrimp lethality test (BSLT) (23, 24). Ten of 48 h post-hatched mature brine shrimps (*Artemia annua* L.) in well-aerated flasks containing 3.8% salinity (w/v) were transferred to petri dishes containing 10 mL of extract concentrations ranging from 0 ppm–5000 ppm. Distilled water was used as a positive control, while sea salt solution as a negative control. After 24 h incubation, mortal shrimps were counted and the percentage of shrimp mortality of each concentration was calculated as follows:

$$\text{Shrimp mortality }(\%)=\frac{\text{Number of killed shrimps}}{\text{Number of transferred shrimps }(\text{i.e.}10)}\times 100$$

The mean of three values of the extract concentration that kills 50% of the shrimp population (LC_50_) was determined by probit regression analysis. All tests were carried out in triplicate on three separate occasions.

#### Haemolytic assay

The haemolytic effect of the extracts on normal human erythrocytes was inspected using a modified method of Evans et al. (25). Approximately 10 mL of blood collected in ethylenediaminetetraacetic acid (EDTA) from an informed consent healthy donor was centrifuged (500× g, 5 min). The tube containing the cell pellet was filled with a 150 mM NaCl solution (pH 7.4) and centrifuged. Next, 1 mL of the cell pellet was transferred to another tube containing 49 mL of 1× PBS to a concentration of 2% haematocrit. Approximately 190 μL of the cell suspension aliquots were added into respective microfuge tubes containing 10 μL of the plant extracts (final concentrations ranging from 1.56 μg/mL–400 μg/mL) and incubated for 45 min at 37 °C on an orbital shaker. Triton-X (20% v/v) in PBS solution (1% final concentration) and 1× PBS were used as positive and negative controls, respectively. The tubes were centrifuged and 100 μL aliquots of the supernatants were transferred into 96-well microtiter plates. The absorbance (haemoglobin concentration) was measured using a microplate reader at 540 nm, and the percentage of haemolysis was calculated as follows:

$$\text{Haemolysis }(\%)=\frac{\text{Absorbance }(\text{test sample}-\text{negative control})}{\text{Absorbance }(\text{positive control}-\text{negative control})}\times 100$$

#### In vitro cytotoxicity assay

The MTT assay was used to determine the cytotoxicity of the extracts (26). NIH/3T3 (normal embryo fibroblast cell) and Vero (normal kidney epithelial cell) cell lines were obtained from the School of Health Sciences and INFORMM, USM, respectively. Both cells were cultured in DMEM medium (Gibco, Waltham, Massachusetts, USA) supplemented with 5% foetal bovine serum (Gibco, Waltham, Massachusetts, USA), and 1% 10,000 U/mL penicillin-streptomycin (Gibco, Waltham, Massachusetts, USA), and incubated at 37 °C in 5% CO_2_. Cells that achieved 70%–80% confluency were trypsinised, seeded at a density of 5 × 10^4^ cells/mL and incubated overnight with 100 μL of media in 96-well microtiter plates at 37 °C in 5% CO_2_. After reaching 70%–80% confluency, the cells were treated with different concentrations of the extracts ranging from 1.56 μg/mL–400 μg/mL and incubated for 72 h at 37 °C. Artemisinin was used as a standard control and 100% DMSO- and only media-containing cells were used as positive and negative controls, respectively. Medium without cells was used as a blank. After treatment, each well was added with 50 μL of MTT tetrazolium salt solution (0.4 mg/mL final concentration, Invitrogen, Waltham, Massachusetts, USA) before incubation for 4 h. All media were carefully discarded, leaving only purple crystal formazan products at the bottom of the wells. A 200 μL solution of 100% DMSO was added to each well and gently mixed using an orbital shaker for 30 min to solubilise the formazan. Absorbance was measured by a microplate reader at 570 nm. The percentage of cell survival was calculated by using the following formula and obtained from three independent experiments.

$$\text{Cell survival }(\%)=\frac{\text{Absorbance }(\text{test sample}-\text{blank})}{\text{Absorbance }(\text{negative sample}-\;\text{blank})}\times 100$$

The mean of three half-maximal cytotoxicity concentration (CC_50_) values of the extracts was determined using GraphPad Prism software (Version 6). The SI was also calculated using a ratio of the CC_50_ to the IC_50_ obtained from the antimalarial assay.

#### Statistical analysis

The data were expressed as mean ± standard deviation (SD) from at least three independent experiments. Data from the in vitro antimalarial and cytotoxicity assays were analysed with the help of computerised GraphPad Prism software (Version 6) to determine the IC_50_ and CC_50_ values, respectively. The data were tested for normality before analysed via one-way analysis of variance (ANOVA), followed by Tukey pairwise comparisons at 95% confidence (comparison between the treatment groups) using Minitab 17. Value of *P* < 0.05 was considered to be statistically significant.

## Results

### Extraction Yield of the Q. infectoria Crude Extracts

The extraction yield (%) recorded is illustrated in Table 1 based on the nature of the solvents used. The methanol extract produced the highest yield (51.64% of the dry powder), followed by the acetone extract (50.85%), the ethanol extract (46.47%) and the aqueous extract (44.85%).

### The Antimalarial Activity of the Crude Extracts Against 3D7 Parasites

The antimalarial activity of the four extracts was evaluated against the 3D7 parasites and summarised in Table 2 and Supplementary material 1. According to WHO guidelines and Lekana-Douki et al. (27), IC_50_ < 15 μg/mL is considered as a promising activity, 15–50 μg/mL as low activity and > 50 μg/mL as inactive. The acetone and methanol extracts exhibited promising antimalarial activity, with the highest antimalarial activity shown by the acetone extract. Meanwhile, the ethanol and aqueous extracts showed low antimalarial activity. *P. falciparum* was highly susceptible to artemisinin, the standard antimalarial drug used in this study. The significant antimalarial activity was demonstrated between all extracts and artemisinin (*P* < 0.05).

### The Toxicological Activity of the Crude Extracts on Brine Shrimps

The toxicological activity of the four extracts against the brine shrimps is displayed in Table 3 and Supplementary material 2. According to Meyer’s and Clarkson’s toxicity index, LC_50_ < 1000 ppm is considered as toxic, and LC_50_ > 1000 ppm as non-toxic (28, 29). All extracts showed an LC_50_ value of > 1000 ppm. This suggests that the extracts were non-toxic to the brine shrimps.

### The Haemolytic Activity of the Crude Extracts on Human Erythrocytes

**Figure 1:**
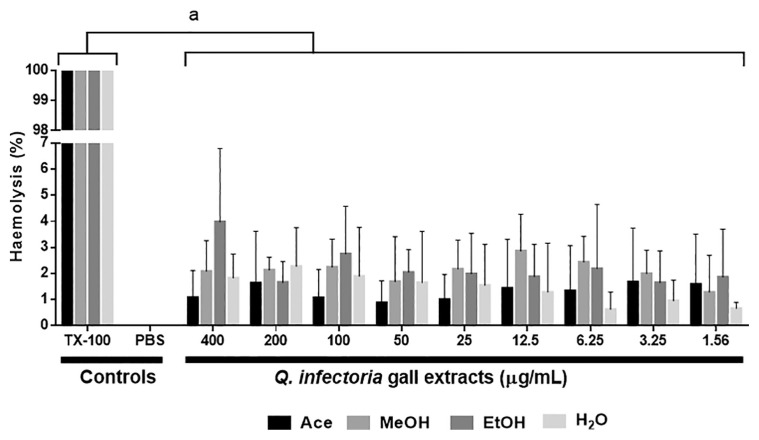
The haemolytic effect of the *Q. infectoria* crude extracts on normal fresh erythrocytes (*n* = 4) after 45 min of incubation. The data represent the mean (SD) of three independent experiments of different blood groups (A^+^, B^+^, O^+^, AB^+^). Mean values were tested for normality before proceeding to the parametric test; one-way ANOVA followed by Tukey multiple comparisons at 95% confidence. Triton-X in 1% (TX-100) and PBS were used as positive and negative controls, respectively. Value of *P* < 0.05 was considered to be statistically significant. The haemolytic activity of the gall extracts was not concentration-dependent. Letter ‘a’ indicates that there was statistical significance between the mean values of the gall extracts (concentrations between 1.56 μg/mL–400 μg/mL) with the positive control. No statistical significance was shown in all extracts when compared with the negative control

The haemolytic activity of the four extracts on human erythrocytes (type A^+^, B^+^, AB^+^, O^+^) is presented in Figure 1. The haemolytic activity of all extracts was not concentration-dependent. According to Choi et al. (30), > 5% haemolysis indicated that the gall extracts cause damage to the erythrocytes. In this study, all extracts caused less than 5% haemolysis compared to the positive control using 1% Triton X-100, which caused 100% haemolysis of the erythrocytes (*P* < 0.05). This indicates that the extracts have no haemolytic effect. No statistical significance was shown in all extracts when compared with the negative control, 1× PBS.

### The Cytotoxicity of the Crude Extracts on Normal Cell Lines

According to the cytotoxicity standard by Haddad et al. (31) and Kweyamba et al. (32), CC_50_ of < 1 μg/mL, 1 μg/mL–10 μg/mL, 10 μg/mL–30 μg/mL and > 30 μg/mL is indicated as high, moderate, mild and non-toxic, respectively. As summarised in Table 4 and Supplementary materials 3(a) and 3(b), all extracts have mild toxicity to the NIH/3T3 cells and non-toxic to the Vero cells. Artemisinin was non-toxic to both cells. The SI (CC_50_/IC_50_), which is defined as a ratio of the cytotoxicity on the NIH/3T3 and Vero cells to the antimalarial activity, was calculated. A low SI indicates that the extract does not only have selectivity towards the malaria parasite but also towards the host, which is detrimental to the host (cells) more than the malarial parasite. In contrast, high SI indicates that the extract has more selectivity towards the malarial parasite, which eventually kills the parasite but not the cells. According to Kwansa-Bentum et al. (33), SI = 2 is the minimum value to validate a safe antimalarial use. As shown in Table 5, the acetone extract has good selectivity for the malaria parasite, with the highest SI values, followed by the methanol extract.

## Discussion

The efficacy of *Q. infectoria* galls against protozoan parasites such as *Leishmania major*, *Blastocystis hominis* and *Entamoeba histolytica* have been confirmed in vitro and in vivo (11, 13–15). To the best of our knowledge, *Q. infectoria* galls have not been studied for its antimalarial activity. Hence, the present study aims to screen the antimalarial potential of *Q. infectoria* gall extracts as a novel resource for antimalarial treatment without compromising its toxicological aspects.

Four polar solvent extracts of *Q. infectoria* galls were tested against *P. falciparum*. The polar solvents with high polarity index, aqueous (P’ = 9), followed by ethanol (P’ = 5.2), methanol (P’ = 5.1) and acetone (P’ = 5.1) were used due to their ability to extract a broad spectrum of bioactive compounds present in the plant. This was confirmed by Muñoz et al. (34) in the search for antimalarial compounds from Bolivian plants using several polar solvents. Our findings exhibited that the acetone and methanol extracts of *Q. infectoria* galls had promising antimalarial activities. This is in agreement with the study by Bagavan et al. (35) in which both acetone and methanol extracts of *Phyllanthus acidus* leaves exhibited a promising antimalarial activity in vitro against the 3D7 strain of *P. falciparum*. This could be explained by the high content of phenolic compounds possessing antimalarial activity associated with these extracts (36, 37). In another study, phenolic compound-rich acetone and methanol extracts of *Q. infectoria* galls also showed a promising antibacterial activity compared to other extracts (38), suggesting that the high content of phenolic compounds in *Q. infectoria* galls are attributable to the antibacterial or antimalarial activity (39) despite the presence of variations in terms of the phenolic composition in the extracts.

The presence of phenolic compounds in *Q. infectoria* galls have been extensively quantified, with 50%–70% tannic acid, gallic acid and ellagic acid as principal constituents (7, 40). Among these phenolic compounds, ellagic acid has been widely studied exhibiting promising antimalarial activities against *P. falciparum* in vitro (41), *P. vinckei* in vivo (41), *P. yeolli* in vivo (42) and *P. berghei* in vivo (43) without any toxicity (42). Moreover, ellagic acid is more active at the mature stage of the parasites (44) during which most of the haemoglobin-rich host cell cytoplasm is ingested and digested.

Next, the toxicological activity of the *Q. infectoria* gall extracts was assessed as the toxicity effect of the plant has not been reported to date. Brine shrimp lethality test (BSLT) was used as a preliminary assay to screen the toxicity of the extracts after 24 h exposure to a simple organism, brine shrimp (45, 46). This assay is economical and utilises a small amount of test material (47). Furthermore, the development of brine shrimp is robust, and the observation of shrimp survival or mortality using a magnifying glass makes this assay convenient (45). According to Zeng et al. (48), brine shrimp domain shares ~83% identity with the human domain, making it a prominent model organism for test purposes. Our study reported that the *Q. infectoria* gall extracts prepared using the polar solvents and subsequently diluted with absolute DMSO had a non-toxic effect on brine shrimps. Therefore, further studies on the toxicity of the *Q. infectoria* gall extracts are necessary to provide more details on their safety.

The toxicological activity of the *Q. infectoria* gall extracts was further explored by observation of their haemolytic effect on normal human erythrocytes. Erythrocytes are one of the vital components in the blood circulation system responsible for supplying oxygen to human tissues in the body. Given the promising effect of the extracts on parasite-infected erythrocytes, it is not known if the extracts even affect normal and uninfected erythrocytes. Overall, all the extracts were non-toxic to the normal erythrocytes. The phenolic compounds in the extracts might act as potent antioxidants that contribute significantly to the antihaemolytic action (49). Antioxidants can counteract the harmful effect of oxidation via a chain-breaking mechanism by neutralising free radicals and subsequently eliminating the radical oxidative scavenging agents by quenching the chain-initiating catalytic agent (50). The correlation between the phenolic compounds and the antioxidant activity of the *Q. infectoria* gall extracts is in line with the previous study showing an excellent quenching ability of the ethanol extract of this plant against 2,2-diphenyl-1-picryl-hydrazyl-hydrate (DPPH) free radicals at a very low IC_50_ value (51). Therefore, the *Q. infectoria* gall extracts are capable of protecting against oxidative damage to lipid bilayer on the erythrocyte membrane.

The cytotoxicity test using tissue cells in vitro is pivotal to predict the potential toxic effect of the plant extract in animals. In this study, fibroblasts and kidney cell lines derived from animal cells were selected based on their characteristics that resemble human cells that are critically important for tissue repair. Cytotoxicity test using MTT assay showed that the *Q. infectoria* extracts caused mild toxic to non-toxic effects for both normal cell lines. The cytotoxicity profile of *Q. infectoria* in our study is in good agreement with those reported by others as non-toxic using a similar assay with other types of cells (11, 52, 53). The major compounds in the extracts, tannins or specifically tannic acid, as reported from previous studies (54, 55), are implied to be the contributor for the low cytotoxicity through its ability to prevent the free-radical-mediated disorders such as inflammation (12, 56), hepatotoxicity (57), lesions in the stomach (58) and enhances the wound healing (59). Tannins are also documented to help protect the galls against pathogens, insects and the other competing plant (60), which are likely to cause the mild toxicity of the extracts to the fibroblast cells. Other combination of the bioactive constituents in this gall extracts may also possess antagonistic, additive or synergistic properties that impart the toxic or non-toxic activities to the mammalian cells (61). Overall, the cell growth in both cell lines was unaffected by the extracts through its ability in maintaining cell integrity (62), highlighting its possible use in the new drug development. Concurrently, both methanol and acetone extracts have promising antimalarial activity, as well as good selectivity compared to the other extracts. Therefore, these extracts are preferable for further research.

## Conclusion

In conclusion, this in vitro study demonstrates a promising antimalarial activity of the acetone and methanol crude extracts of the *Q. infectoria* galls against *P. falciparum.* All the crude extracts are non-toxic to the brine shrimps and normal erythrocytes. The acetone and methanol extracts are also nontoxic to the normal cell lines with acceptable SI. These findings will lead to further studies on the bioactivity-guided isolation of bioactive compounds from the acetone and methanol extracts of the *Q. infectoria* galls.

## Supplementary Information

Supplementary material 1 Log concentration-response curve of the *Q. infectoria* crude extracts (A) and artemisinin (B) against the chloroquine-sensitive (3D7) strain of *P. falciparum*. The horizontal dashed line corresponds to the approximate mean IC_50_ value after extrapolating with the *x*-axis

Supplementary material 2 The mean LC_50_ value (SD) of the *Q. infectoria* crude extracts against the brine shrimps. The horizontal dashed line corresponds to toxicity baseline. The values of **P < 0.01 and **P < 0.001 using one-way ANOVA indicate significant differences between the treatment groups

Supplementary material 3(a) Log concentration-response curve of the *Q. infectoria* crude extracts on fibroblast cell (NIH/3T3) (A) and kidney cell lines (Vero) (B). The horizontal dashed line corresponds to the approximate mean CC_50_ value after extrapolating with the *x*-axis

Supplementary material 3(b) Log concentration-response curve of artemisinin on fibroblast cell (NIH/3T3) and kidney cell lines (Vero). The horizontal dashed line corresponds to the approximate mean CC_50_ value after extrapolating with the *x*-axis

## Figures and Tables

**Table 1:** The extraction yield (%) of the *Q. infectoria* crude extracts prepared using different polar solvents

Solvent	Yield of the extract (%)
Acetone	50.85
Methanol	51.64
Ethanol	46.47
Aqueous	44.85

**Table 2:** The antimalarial activity of the *Q. infectoria* crude extracts

Extract	Antimalarial activity against the malaria parasite, IC_50_ (μg/mL)	*F*-statistics (DFn, DFd)	*P*-value
Acetone	5.85 (1.64)	F (4, 10) = 289.696	*P* < 0.0001
Methanol	10.31 (1.90)		
Ethanol	20.00 (1.57)		
Aqueous	30.95 (1.27)		
Artemisinin	0.005 (0.001)		

Notes: The data were expressed as mean (SD) of three independent experiments. Mean values were tested for normality before proceeding to the parametric test; one-way ANOVA followed by Tukey multiple comparisons at 95% confidence. Value of *P* < 0.05 was statistically significant. Acetone, methanol, ethanol and aqueous were known as treated groups. Artemisinin was known as a control group. The acetone was statistically significant as compared with the methanol (*P* = 0.0004), ethanol (*P* < 0.0001), aqueous (*P* < 0.0001) and artemisinin (*P* = 0.0035). The methanol was statistically significant as compared with the acetone (*P* = 0.0004), ethanol (*P* < 0.0001), aqueous (*P* < 0.0001) and artemisinin (*P* < 0.0001). The ethanol was statistically significant as compared with the acetone (*P* < 0.0001), methanol (*P* < 0.0001), aqueous (*P* < 0.0001) and artemisinin (*P* < 0.0001). The aqueous was statistically significant as compared with the acetone (*P* < 0.0001), methanol (*P* < 0.0001), ethanol (*P* < 0.0001) and artemisinin (*P* < 0.0001). DFn = degree of freedom numerator; DFd = degree of freedom denominator

**Table 3:** The toxicological activity of the *Q. infectoria* crude extracts against brine shrimps

Extract	Toxicological activity on the brine shrimp, LC_50_ (ppm)	*F*-statistics (DFn, DFd)	*P*-value
Acetone	1802.44 (134.18)	*F* (3, 8) = 25.92	*P* = 0.0002
Methanol	1321.08 (97.27)		
Ethanol	1966.78 (93.60)		
Aqueous	1325.19 (120.46)		

Notes: The data were expressed as mean (SD) of three independent experiments. Mean values were tested for normality before proceeding to the parametric test; one-way ANOVA followed by Tukey multiple comparisons at 95% confidence. Value of *P* < 0.05 was statistically significant. The acetone was statistically significant as compared with the methanol (*P* = 0.0035) and aqueous (*P* = 0.0037). The ethanol was statistically significant as compared with the methanol (*P* = 0.0005) and aqueous (*P* = 0.0005). No statistical significance between acetone and ethanol and between methanol and aqueous. DFn = degree of freedom numerator; dFd = degree of freedom denominator

**Table 4:** The toxicological activity of the *Q. infectoria* crude extracts on the normal cell lines

Cells	Toxicological activity on the normal cell lines, CC_50_ (μg/mL)					*F*-statistics (DFn, DFd)	*P*-value

	Acetone	Methanol	Ethanol	Aqueous	Artemisinin		
NIH/3T3	23.60 (1.18)	20.67 (1.19)	21.88 (1.03)	24.14 (1.32)	111.43 (1.41)	*F* (4, 10) = 13.347	*P* = 0.0005
Vero	39.32 (1.34)	34.75 (1.07)	42.00 (1.11)	39.51 (1.68)	198.73 (3.13)	*F* (4, 10) = 59.763	*P* < 0.0001

Notes: The data were expressed as mean (SD) of three independent experiments. Mean values were tested for normality before proceeding to the parametric test; one-way ANOVA followed by Tukey pairwise comparisons at 95% confidence. Value of *P* < 0.05 was statistically significant. Acetone, methanol, ethanol and aqueous were known as treated groups. Artemisinin was known as a control group. No statistical significance was shown between all treated groups for both normal cell lines. For NIH/3T3 cells, the acetone, methanol, ethanol and aqueous were statistically significant as compared with the artemisinin (*P* = 0.0014; *P* = 0.0011; *P* = 0.0012; *P* = 0.0015). For Vero cells, the acetone, methanol, ethanol and aqueous were statistically significant as compared with the artemisinin (*P* < 0.0001; *P* < 0.0001; *P* < 0.0001; *P* < 0.0001). DFn = degree of freedom numerator; DFd = degree of freedom denominator

**Table 5:** The SI of the *Q. infectoria* extracts on embryo fibroblast (NIH/3T3) and kidney epithelial cell lines (Vero)

Extract	SI	

	NIH/3T3	Vero
Acetone	4.0	6.7
Methanol	2.0	3.4
Ethanol	1.1	2.1
Aqueous	0.8	1.3
Artemisinin	> 200.0	> 200.0
