# Supplementary figures and images for: In Vitro Antimalarial and Toxicological Activities of Quercus infectoria (Olivier) Gall Extracts
Source: Malays J Med Sci. 2020 Aug 19;27(4):36–50. doi: 10.21315/mjms2020.27.4.4 (PMC7444841; doi:10.21315/mjms2020.27.4.4)

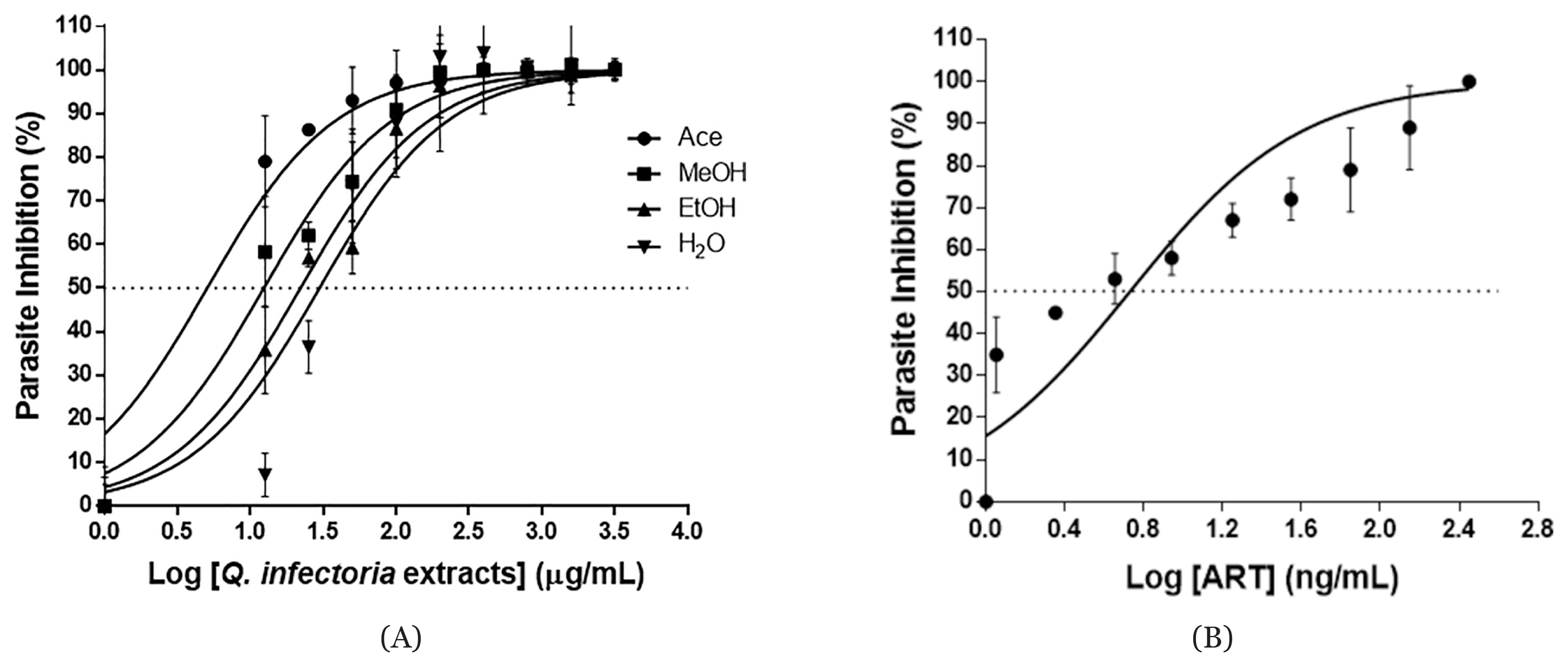

Supplement: Supplementary material 1 — Log concentration-response curve of the Q. infectoria crude extracts (A) and artemisinin (B) against the chloroquine-sensitive (3D7) strain of P. falciparum. The horizontal dashed line corresponds to the approximate mean IC50 value after extrapolating with the x-axis [file 04mjms27042020_oa1s1.tif]

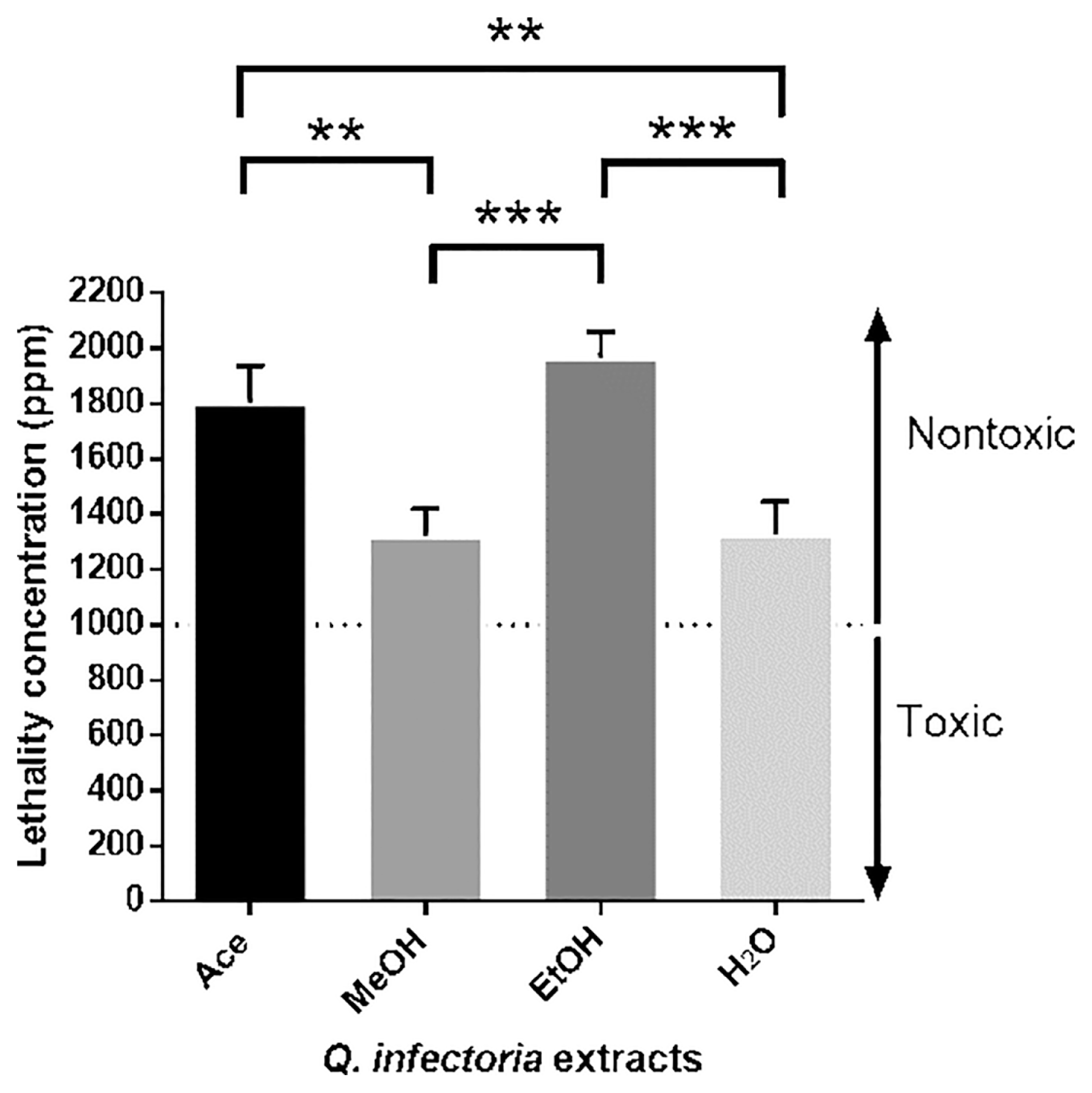

Supplement: Supplementary material 2 — The mean LC50 value (SD) of the Q. infectoria crude extracts against the brine shrimps. The horizontal dashed line corresponds to toxicity baseline. The values of **P < 0.01 and **P < 0.001 using one-way ANOVA indicate significant differences between the treatment groups [file 04mjms27042020_oa1s2.tif]

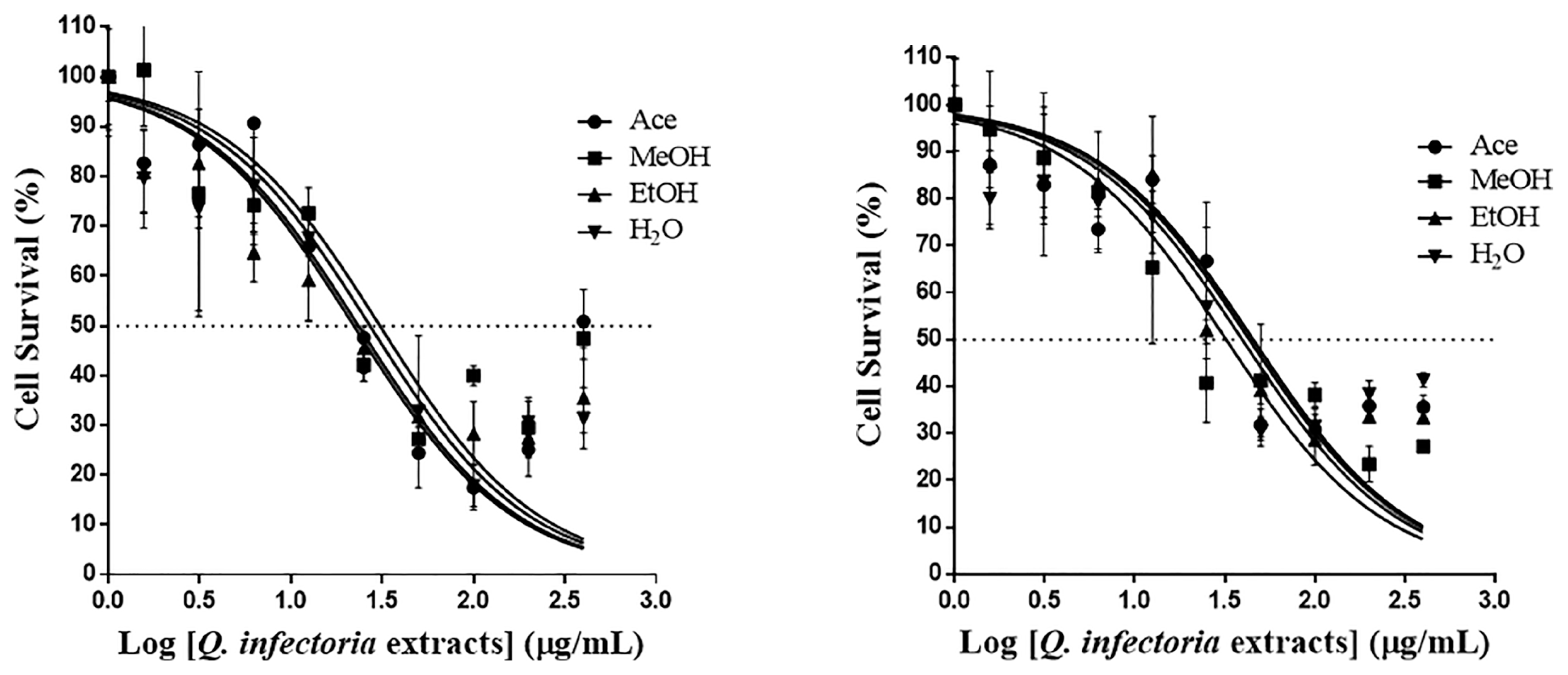

Supplement: Supplementary material 3(a) — Log concentration-response curve of the Q. infectoria crude extracts on fibroblast cell (NIH/3T3) (A) and kidney cell lines (Vero) (B). The horizontal dashed line corresponds to the approximate mean CC50 value after extrapolating with the x-axis [file 04mjms27042020_oa1s3a.tif]

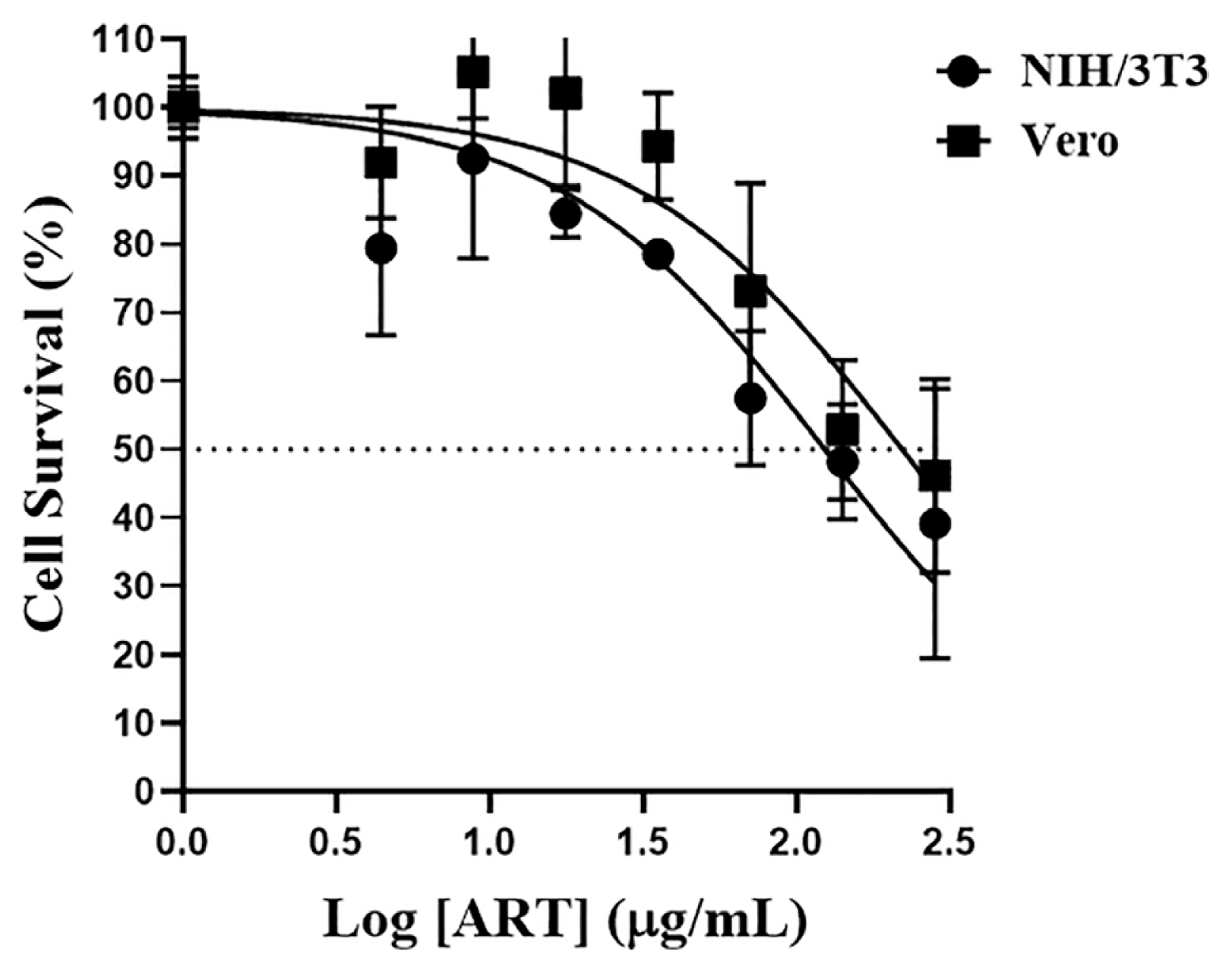

Supplement: Supplementary material 3(b) — Log concentration-response curve of artemisinin on fibroblast cell (NIH/3T3) and kidney cell lines (Vero). The horizontal dashed line corresponds to the approximate mean CC50 value after extrapolating with the x-axis [file 04mjms27042020_oa1s3b.tif]
